# Stone Throwing in the Glass House

**DOI:** 10.3389/fped.2016.00020

**Published:** 2016-03-14

**Authors:** James Anthony Lin

**Affiliations:** ^1^Division of Pediatric Critical Care Medicine, Department of Pediatrics, Mattel Children’s Hospital UCLA, University of California Los Angeles, Los Angeles, CA, USA

**Keywords:** empathy, moral judgment competence, doctor–patient relationship, dehumanization, standard of care, work–life balance

A small girl with a unique genetic syndrome lies in bed since birth. Her brain does not work. She is in a persistent vegetative state. Recurrent seizures punctuate her intractable epilepsy. She shows no meaningful interaction. Her breathing is insufficient. Upper airway obstruction, repeated aspiration events, chronic lung disease, and scoliosis make her struggle to breathe. Her parents never wanted her to suffer. Two years ago, they agreed to a Physician Orders for Life-Sustaining Treatment (POLST). Their daughter would not undergo resuscitation. Death did not come, however. With meticulous medical care, her body grew. Her breathing worsened. Offered either palliation or a tracheostomy tube, her parents chose “trach.” With easier breathing, the girl expended fewer calories. She continued gastrostomy tube feeds and gained 10 pounds in 3 months. Now she is rehospitalized with respiratory distress. Her weight rises another 2 pounds. We put off calorie reduction for the outpatient setting. For the first time, we discharge the family home with a mechanical ventilator. Our collective health-care efforts have set up a protracted course whose likely end will come in overwhelming sepsis or ARDS, rather than allowing natural death with palliative care. How many ethical issues were raised by this case? I count more than 40 (Table 1).

**Table 1:** **Ethical issues raised by the case**.

**Autonomy**
Patient’s autonomy: no independent ability to make choices
Parental autonomy
Difficult choices
Medical
Spiritual or philosophical
Personal
Financial
Mother’s preferences vs. father’s preferences
Evolving attitudes
Gastrostomy tube and artificial feeds
Physician Orders for Life-Sustaining Treatments (POLST)
Treatment of infections
Avoidance of mechanical ventilation and intensive care
Respiratory support *via* bilevel positive airway pressure
Respiratory support *via* non-invasive mechanical ventilation
All care at home, not subacute care facility
Tracheostomy tube instead of palliation
Intensive care
Acquiescence to home mechanical ventilation
Discontinuation of POLST
Physician autonomy
Choices we offer
Gastrostomy tube vs. no gastrostomy tube
Tracheostomy tube vs. palliation
Intensive care vs. no intensive care
Home mechanical ventilator vs. no home mechanical ventilator
No offer of scoliosis treatment
Timing of choices
Withholding certain choices because of patient’s poor prognosis
Delay in therapies associated with longer patient suffering
Choices we make
Avoidance of blood gas evaluations
Allowing weight to rise
Limits of time spent educating and consulting with parents
Native language interpreter for only the most important conversations
**Beneficence**
Support of parents’ psychosocial needs
Maintaining patient’s life while holding out hope for neurologic improvement
Relief of dyspnea
Treatment of infections
Relief of hunger
Intermittent intensive medical care
**Non-maleficence**
Avoidance of neurologic depressive adverse effects of antiepileptics
Patient’s experience of suffering
Inability to express
No vocalization
Non-specific response to pain
Non-specific eye opening
Rare spontaneous smiles
Dyspnea
Constipation
Pain and discomfort of medical procedures
Phlebotomy
Intravenous access
Respiratory suctioning
Parental understanding
Death: physiologic state vs. mysterious ending
Physicians’ consideration of parents’ attachment to child vs. child’s own suffering
**Justice**
Use of intensive care beds
Expenditure of subspecialty personnel effort and time

Physicians make ethical decisions constantly in health care without formal training in moral practice. Consequently, we leave most moral issues unaddressed. Instead, we concretize and categorize patient complaints into physical and mental issues to be addressed functionally (1). For instance, I may not be able to help a boy dying of cancer with his existential loneliness, but I can prescribe morphine for his respiratory distress. As a doctor trained in physiological intervention, this essentialization of clinical questions is justified by the ethical principle of beneficence. We focus on how we can help patients. However, this practice dehumanizes patients and leaves important overarching questions unaddressed. As Agledahl et al. wrote, “Even if your clinically sound decision is morally motivated, it may not necessarily be the morally good thing to do” (1).

What is the effect of practicing medicine without addressing moral issues? Does clinical practice diminish our own humanity? Studies of our medical trainees may shed some light. Remarkably consistent studies have observed that empathy declines in medical students and resident trainees during their clinical years, but not during preclinical education (2). Students are subjected to mistreatment by mentors, loss of idealism when faced with clinical realities, isolation from social supports, high workload, lack of sleep and personal time, and a fragmented patient–physician relationship (2). Furthermore, the evolving electronic medical environment may be reducing trainees’ time spent in patients’ rooms. Pediatric interns and residents now spend only 12% of their time with patients, but 21% on computers and 35% communicating with colleagues (3).

One could argue that limited empathy can improve problem-solving ability and competent health care (4). But what about the moral aspect of medicine? Unfortunately, clinical training impairs moral development. Repeated studies over the past 20 years observed that medical students fail to attain normal moral development and may even decline in moral reasoning capacity during their 4 years of medical school (5–9). Ethics lectures and clinical experience fail to correct this deficiency. One reasonable hypothesis supposes that overemphasis on memorization of facts, obedience to authority, and application of rules based on practice standards, evidence, and cost limits might impede the development of higher order “postconventional,” or ethically principled, thought (7, 8). An evidence-based solution for teaching moral clinical practice is lacking. Despite that, the next time a pediatrics intern struggles with an uncooperative family and wants to spend more time considering the merits of the parents’ arguments, I will quell my inner urge to cut them off in order to move on to the next patient.

Years ago as a sleepless young attending, I paced behind a nursing station. Alarms rang and were silenced, while a faint scent of bleach permeated the air. A previously vivacious toddler, now glassy eyed, was breathless with acute viral bronchiolitis. For the second time, I approached the mother and said, “Your daughter needs help to breathe. We need to place her on a mechanical ventilator.”

Cradling her daughter in her arms, the mother looked directly in my eyes. She said, “No.”

Exhortations by our chief of emergency medicine yielded the same negative response. Against my better judgment, I agreed to trial high flow oxygen in my pediatric intensive care unit. The poor girl struggled all night, lost her intravenous (IV) catheter, and decompensated during attempts to regain IV access. After I finally intubated the child against the wishes of the parents, shortly thereafter the girl suffered cardiac arrest. Two hours of coding with a multidisciplinary team of 20 people was of no use. She was dead. First, I lost faith in parental “autonomy” to make health-care decisions for their children. Worse yet, I then lost faith in my colleagues. While making allowance for my emotionally laden and possibly inaccurate perceptions, I recall uniformly negative feedback from all corners. There was neither support nor empathy.

Why did this happen? Was I treated as less than a person in both the clinical encounter and sequelae? While we discussed above the failure of doctors to treat the whole person, we now focus on how doctors are themselves treated. Dehumanization in medicine goes both ways. People who feel a greater need for medical services recall fewer personal facts about their doctors, want physicians who focus only on their patients, and are less likely to perceive their physicians as having personal attributes or personal lives (10). This leads to the counterproductive result that patients with the greatest medical need are least able to communicate effectively with their most “instrumental” physicians.

A cultural loss of trust in the doctor–patient relationship also plays a role. As famously pointed out by the Institute of Medicine (IOM) (11), deadly medical errors are common. Despite tremendous improvements in US health-care quality since the IOM report (12), popular news continues to trumpet, “Medical errors in Indiana hit another high” (13). Loss-frame messaging (14) undermines the public trust in health-care providers, prejudicing perceptions of incompetence and aloofness.

Physicians ourselves add to the negative chorus. This was formally documented in a standardized covert patient study of consenting physicians (15). In a referral group of oncologists and family physicians, conversations about the other doctor were recorded and analyzed. The large majority of comments – 67% – were Critical, while only 29% were Supportive and 4% Neutral. For most health-care providers, these results should be no surprise. Doctors criticize doctors as lustily as do patients. Nevertheless, I seem to perceive the situation improving recently as doctors place greater emphasis on professionalism.

Some criticism may be understandable, however. Our own “standard of care” is riddled with mistakes. In the decade from 2001 to 2010, a study of 363 original articles testing standard of care found that 146 (40%) reversed accepted medical practices (16). Among these reversals were such entrenched pediatric interventions as imaging studies after first febrile urinary tract infection in young children, high-dose epinephrine for children with in-hospital cardiac arrest, and early insertion of tympanostomy tubes for persistent otitis media >3 months or persistent middle ear effusions. The critical care literature has noted reversals in low-dose dopamine to improve renal blood flow (17), early goal-directed therapy bundles and activated protein C for septic shock (18), gastric residual volumes to guide enteral feeds (19), standard single syringe size for pediatric inotrope infusions (20), and bowel rest for acute pancreatitis (19). A particularly troubling study demonstrated longer waiting times, slower and less use of thrombolysis, and worse mortality for in-hospital than community onset stroke (21). In the last few months of 2015 alone, additional decades-old practices were called into question. Advanced life support (ALS) may be inferior to basic life support (BLS) for out-of-hospital medical emergencies (22). Calcium supplementation may offer no net benefit for bone mineral density or fracture prevention (23, 24). Hypotonic fluids should no longer be used for maintenance fluids (25). One could understand why the public, and physicians ourselves, are confused about whether doctors actually know anything. Thus, the glass house of medicine requires constant rebuilding, not stones cast from within.

The evidence is consistent and clear on deficiencies in physician empathy, moral judgment, and collegial humility. Is this a work–life issue? Do the compromises we make to our core values at work affect our overall personhood? Indeed, yes. This goes beyond feeling sad about a patient death or being inconvenienced by our hectic work schedules. Much is written about what “you” the individual can do to achieve work–life balance, but how we treat each other at work affects all of our lives. At the end of the day, we cannot dismiss our work ills as easily as doffing our white coat. Some dare go so far as to declare our work problems a matter of life and death. In one of the most thorough studies of its kind, a 20-year prospective observational study of 820 healthy employees examined multiple job stresses and found only one overall independent predictor of mortality: peer social support (26). In other words, the way we treat each other at work affects our ultimate outcome measure for clinical studies, mortality. Fortunately, the problems we have created in our health-care system are something we can correct together. Together, we need to remember who we hoped to become when we wrote our medical school application essays. Together, we can strive to show the compassion we feel inside, allow each other to say the word love, and provide the care we yearn to, for our patients and our coworkers. As we heal our patients, we can also heal each other.

A common theme underlay my medical school, pediatric residency, and pediatric critical care fellowship training: “You never learn so much as when you’re screwed.” Putting medical students and trainees on the spot with pimping and publicly pronouncing negative feedback were embraced. As medical educators, mentors, and colleagues in the modern era we now must teach and behave differently from how we learned. What was once malignant needs to be replaced with understanding and care. Collegiality must replace competition. Expressions of gratitude benefit both the recipient and the person who expresses it (27). Incidentally, the same principles apply to how we treat our families outside of work. The happiest families remember to thank each other (28), and the happiest lives are lived in love (29). Even though physicians’ work–life balance may be dominated by our work, one of our most important clinical responsibilities is to help each other be and live well.

## Author Contributions

JL conceived of the idea for this manuscript, conducted the literature review, and drafted the manuscript.

## Conflict of Interest Statement

The author declares that the research was conducted in the absence of any commercial or financial relationships that could be construed as a potential conflict of interest.

## Abbreviations

ALS, advanced life support; ARDS, acute respiratory distress syndrome; BLS, basic life support; IOM, Institute of Medicine; IV, intravenous.
